# Supplementary material for: Identification of photoperiod-induced specific miRNAs in the adrenal glands of Sunite sheep (Ovis aries)
Source: Front Vet Sci. 2022 Jul 22;9:888207. doi: 10.3389/fvets.2022.888207 (PMC9354845; doi:10.3389/fvets.2022.888207)
Supplement: Supplementary file 1 [file Data_Sheet_1.ZIP › Supplementary file/miRDeep2 file.zip!/miRDeep2 file/Novel_Structure/SPLP42A3/Novel_470.pdf]

Provisional ID	:	NC_019484.2_39160
Score total	:	89.9
Score for star read(s)	:	3.9
Score for read counts	:	82.7
Score for mfe	:	1.7
Score for randfold	:	1.6
Score for cons. seed	:	
Total read count	:	174
Mature read count	:	170
Loop read count	:	0
Star read count	:	4

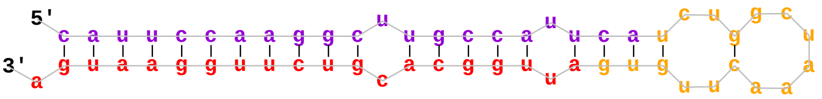

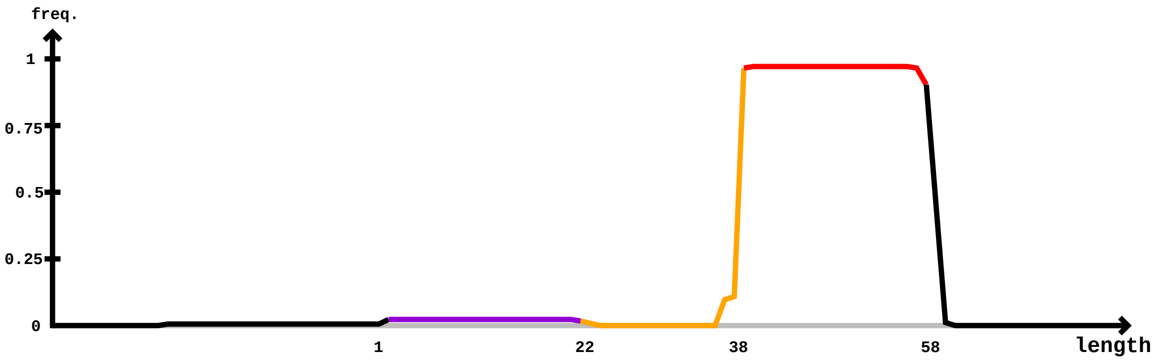

Star Mature

```
5'- uugccaccuucagcgugucugugugcuguaccccauuccaaggcuugccauucaucuggcuaaacuugugauuggcacgucuuggaaugaaguaacguacaacaaugaug   -3'    obs
    uugccaccuucagcgugucugugugcuguacccauuccaaggcuugccauucaucuggcuaaacuugugauuggcacgucuuggaaugaaguaacguacaacaaugaug          exp
    ..((........))..((((((((((..(((..(((((((((((.(((((.((((..(......)..)))).))))).)))))))))))..)))..)))).)))..))).     reads      mm        sample
    ..........cagcgugucugugugcuguaccc.............................................................................     1          0         seq
    .................................cauuccaaggcuugccauuc.........................................................     1          0         seq
    .................................cauuccaaggcuugccauuca........................................................     3          0         seq
    ....................................................................ugauuggcacgucuuggaau......................     1          0         seq
    ....................................................................ugauuggcacgucuuggaauga....................     14         0         seq
    ....................................................................ugauuggcacgucuuggaaugaa...................     2          0         seq
    .....................................................................gauuggcacgucuuggaaug.....................     1          0         seq
    .....................................................................gauuggcacgucuuggaauga....................     1          0         seq
    ......................................................................auuggcacgucuuggaaug.....................     10         0         seq
    ......................................................................auuggcacgucuuggaauga....................     107        0         seq
    ......................................................................auuggcacgucuuggaaugaa...................     31         0         seq
    ......................................................................auuggcacgucuuggaaugaag..................     2          0         seq
    .......................................................................uuggcacgucuuggaauga....................     1          0         seq
```
